# Current status and characteristics of work-related musculoskeletal disorders among general surgeons in japan: a cross-sectional survey at a university hospital and its affiliated regional hospitals

**DOI:** 10.1007/s00595-025-03174-z

**Published:** 2025-11-14

**Authors:** Hideki Sasanuma, Hiroshi Kawahira, Hironori Yamaguchi, Joji Kitayama, Naohiro Sata

**Affiliations:** 1 Division of Gastroenterological, General and Transplant Surgery, Department of Surgery, Jichi Medical University, 3311-1 Yakushiji, Shimotsuke, 329-0498 Tochigi Japan; 2 Medical Simulation Center, Jichi Medical University, 3311-1 Yakushiji, Shimotsuke, 329-0498 Tochigi Japan

**Keywords:** Surgeons, Musculoskeletal disorders, Neck pain, Nonsteroidal anti-inflammatory drugs, Minimally invasive surgery

## Abstract

**Purpose:**

There is limited awareness of work-related musculoskeletal disorders (MSDs) in Japan, despite the high ergonomic risks for surgeons. We conducted this study to investigate the prevalence, characteristics, and impact of MSDs on Japanese general surgeons.

**Methods:**

An electronic survey of 136 general surgeons at a Japanese university hospital network used a modified Nordic Musculoskeletal Questionnaire to assess demographics, work factors, MSD symptoms, psychological distress, use of nonsteroidal anti-inflammatory drugs (NSAIDs), and their impact.

**Results:**

Based on a 56.6% response rate, we found a high prevalence of chronic (37.7%) and acute (51.9%) MSDs. These disorders frequently impacted surgeons’ work (30.0%) and daily life (39.0%), leading to time off (5.2%) and medical intervention (28.6%). Both MSD types correlated significantly with the use of NSAIDs and psychological distress. Notably, neck pain was strongly associated with the use of NSAIDs. The proportion of minimally invasive surgical procedures performed each week was associated significantly with acute, but not chronic, MSDs.

**Conclusions:**

MSDs are highly prevalent among Japanese surgeons, impacting their physical and psychological health. The high symptom prevalence and the strong association between neck pain and NSAID reliance underscore the urgent need for ergonomic interventions and preventive strategies in surgical practice to protect this essential workforce.

**Supplementary Information:**

The online version contains supplementary material available at 10.1007/s00595-025-03174-z.

## Introduction

Work-related musculoskeletal disorders (MSDs) represent a significant occupational health challenge across numerous professions, particularly those involving repetitive movements and prolonged physical exertion. Surgeons face an especially elevated risk because of the inherent physical demands of their work, which necessitates maintaining static postures for extended periods, applying force, and performing repetitive tasks in the operating theater [1]. The increasing adoption of minimally invasive surgical techniques has brought the prevalence of MSDs among surgeons into sharper focus [2]. Notably, the anatomical sites affected by MSDs vary between open and minimally invasive surgery [3], but the impact of MSDs on surgeons is substantial, affecting not only their health but also their professional longevity and performance. Many surgeons require medical intervention for MSD-related conditions, which have been associated with burnout, career dissatisfaction, and early retirement. Furthermore, MSDs can compromise surgical performance and patient safety, with significant consequences for individuals, healthcare institutions, and society.

In Japan, awareness among general surgeons remains limited, despite some professional societies issuing recommendations for specialists [4] and studies in Western countries documenting high MSD prevalence rates [5]. Comprehensive epidemiological data are lacking amidst systemic challenges such as long working hours and staff shortages; issues which the nationwide physician work-style reform initiated in 2024 aims to address [6, 7]. Recent nationwide surveys have highlighted the ongoing significant disparities in working conditions and satisfaction among surgical trainees [8]. These challenges contribute to a projected decline in the number of general surgeons [9–11], making the understanding of MSDs critical for workforce sustainability. To date, research on this topic in Japan has been limited to a single foundational report by Owada et al. [12]. While this study confirmed the high prevalence of MSDs and suggested that open surgery posed a greater risk, key questions about the consequences and specific risk factors in the modern surgical environment remain unanswered.

The primary aims of this study were threefold: first, to provide updated MSD prevalence data in the context of these ongoing work-style reforms; second, to quantitatively assess the link between MSDs and critical outcomes such as psychological distress and reliance on nonsteroidal anti-inflammatory drugs (NSAIDs); and third, to investigate the complex ergonomic impact of high-volume minimally invasive surgery. By addressing these specific gaps, we aimed to provide a robust evidence base to guide the development of targeted ergonomic interventions to safeguard the health of this essential workforce.

## Materials and methods

This study employed a cross-sectional survey design and was conducted in accordance with the principles of the Declaration of Helsinki. The research protocol was reviewed by the Institutional Review Board of Jichi Medical University Hospital (Approval No. 25 − 007) and was determined to be exempt from formal ethical review.

### Setting

The study was conducted at Jichi Medical University Hospital and its affiliated regional hospitals in Tochigi Prefecture and neighboring areas. The Department of Surgery at the university dispatches personnel to these key regional hospitals to support local surgical care.

### Participants

The study targeted all 136 general surgeons registered on the departmental mailing list and working at the participating institutions as of April, 2024. Eligibility criteria included being a general surgeon employed at the university hospital or one of its affiliated regional hospitals during the survey period (July to September, 2024). Participation was voluntary and anonymous; the return of a completed questionnaire was considered as implied consent.

### Questionnaire and survey administration

The questionnaire used in this study, consisting of 29 questions, was based on the Nordic Musculoskeletal Questionnaire (NMQ) (see Supplementary Fig. 1 and Supplementary Table 1) [13]. The original NMQ was translated into Japanese by two independent native Japanese-speaking medical professionals. This translated version was then reviewed by a panel of surgeons and experts in musculoskeletal health to ensure linguistic and cultural validity. It was subsequently edited and modified for clarity and relevance to the target population. The web-based survey was created using Microsoft Forms (Microsoft Corp., Redmond, WA, USA), with an estimated response time of 5 min. The survey was conducted from July to September, 2024, and the survey period was standardized across all participating institutions.

### Primary outcome

The primary outcomes were the prevalence of chronic and acute musculoskeletal disorders (MSDs). Based on responses to the NMQ, ‘chronic MSDs’ were defined as MSDs persisting for more than 12 months, and ‘acute MSDs’ were defined as MSDs experienced within the past 7 days.

### Factors

We collected data on potential risk factors through the questionnaire. These included:

- Surgeon background factors: Age, gender, height, weight, and years of work experience.
- Work-related factors: Weekly hours of surgery, duration of the longest operation in a week, and the weekly percentage of minimally invasive surgery (MIS), where MIS was defined to include both laparoscopic and robotic-assisted procedures. In our study cohort, robotic-assisted surgery was performed by a limited number of surgeons exclusively at the university hospital; therefore, the case volume was insufficient for a separate statistical sub-analysis.
- Other factors: The questionnaire also assessed the impact of MSDs on surgical practice and daily life, the use of NSAIDs, and associated psychological distress.

### Data treatment and statistical analysis

Non-responses were treated as missing data. For analysis, quantitative variables were categorized as needed. For example, work experience was grouped into 10-year increments, and the weekly proportion of MIS was grouped into 20% increments. Similarly, variables for weekly and longest operating times were dichotomized for association testing (for example, > 10 h vs. ≤10 h).

Data were analyzed using JMP Pro 17.2.0 (SAS Institute Inc., Cary, NC, USA). Chi-square tests and Fisher’s exact test were used to examine associations between categorical variables. To evaluate the subjective impact of different surgical modalities, respondents who reported MSDs were asked to rate the severity of their symptoms attributable to open surgery and, separately, to laparoscopic surgery. The severity was rated on a 4-point Likert scale (1 = none, 2 = mild, 3 = moderate, 4 = severe), as detailed in Supplementary Table 1. The relationship between these two ordinal ratings was then assessed using Spearman’s rank correlation coefficient. Additionally, nominal logistic regression analysis was conducted to examine the association between NSAIDs use and MSDs-affected sites, with models adjusted for potential confounding factors such as age and gender. A P-value < 0.05 was considered significant.

## Results

**Fig. 1:**
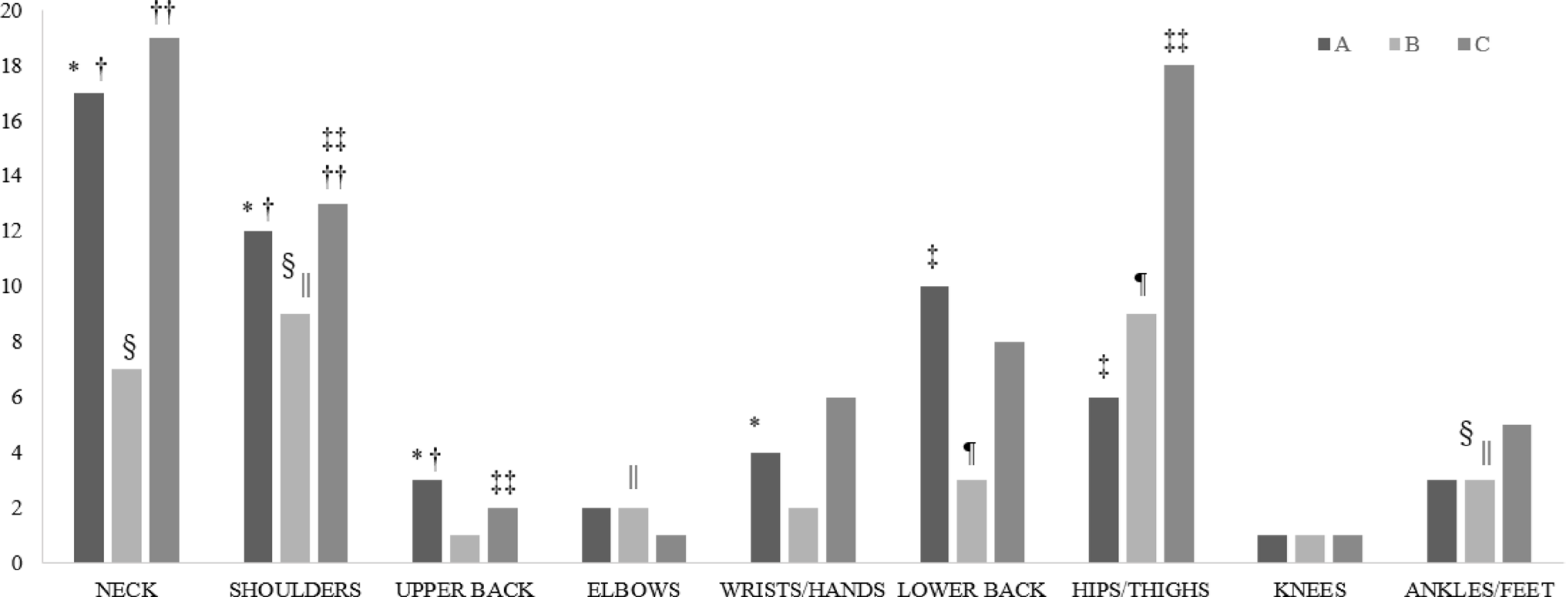
Location-specific number of musculoskeletal disorders (MSDs). The bar graph represents the frequency of musculoskeletal disorders (MSDs) across different body regions, categorized as follows: Group A (chronic MSDs, defined as persistent issues during the last 12 months), Group B (activity limitation caused by issues in the last 12 months), and Group C (acute MSDs, defined as an issue experienced in the last 7 days). The x-axis shows body locations from the neck to ankles/feet, and the y-axis indicates the number of reported disorders. Different statistical symbols (*, †, ‡, §, ||, ¶, ⁑, ††, ‡‡) represent significant differences between the groups (*P* < 0.05). Neck, shoulders, and lower back show the highest frequency of MSDs across all three groups. MSDs: musculoskeletal disorders

The survey was completed by 77 out of 136 respondents (56.6%), with an average response time of 4 min and 45 s. Table 1 summarizes the backgrounds and work-related characteristics of the respondents. Figure 1 illustrates the location-specific prevalence of MSDs according to the three main components of the NMQ: an issue experienced in the past 12 months (A), the prevention by this issue of normal activities in the past 12 months (B), and an issue experienced in the past 7 days (C). Twenty-nine surgeons (37.7%) reported chronic MSDs, with each surgeon experiencing up to five MSD locations. The highest incidence was observed in the neck (*n* = 17, 22.1%), followed by the shoulders (*n* = 12, 15.6%) and lower back (*n* = 10, 13.0%). Furthermore, 22 surgeons (28.6%) reported that chronic MSDs had affected their work. Acute MSDs were reported by 40 surgeons (51.9%), with a maximum of four affected locations per individual. The most common areas were the neck (*n* = 19, 24.7%), hip/thigh (*n* = 18, 23.4%), and shoulders (*n* = 13, 16.9%).

The impact of these conditions was substantial. For example, 23 surgeons (30.0%) stated that MSDs had negatively impacted their surgical practice, and 4 (5.2%) had taken a leave of absence as a result. Thirty surgeons (39.0%) reported that their daily life was adversely affected, and 22 (28.6%) had sought medical attention. Regular use of NSAIDs was reported by 29 surgeons (37.7%), with 15 (19.5%) taking them at least once per month. A significant correlation was found between the use of NSAIDs and both chronic MSDs (Pearson’s χ^2^ = 33.65, *P* < 0.001) and acute MSDs (Pearson’s χ^2^ = 13.39, *P* < 0.001). Furthermore, 41 surgeons (53.2%) attributed their MSDs to work-related factors, while 44 (57.1%) associated them with psychological distress. A significant correlation was observed between mental distress and both chronic MSDs (Pearson’s χ^2^ = 18.66, *P* < 0.001) and acute MSDs (Pearson’s χ^2^ = 6.77, *P* = 0.009).

**Fig. 2:**
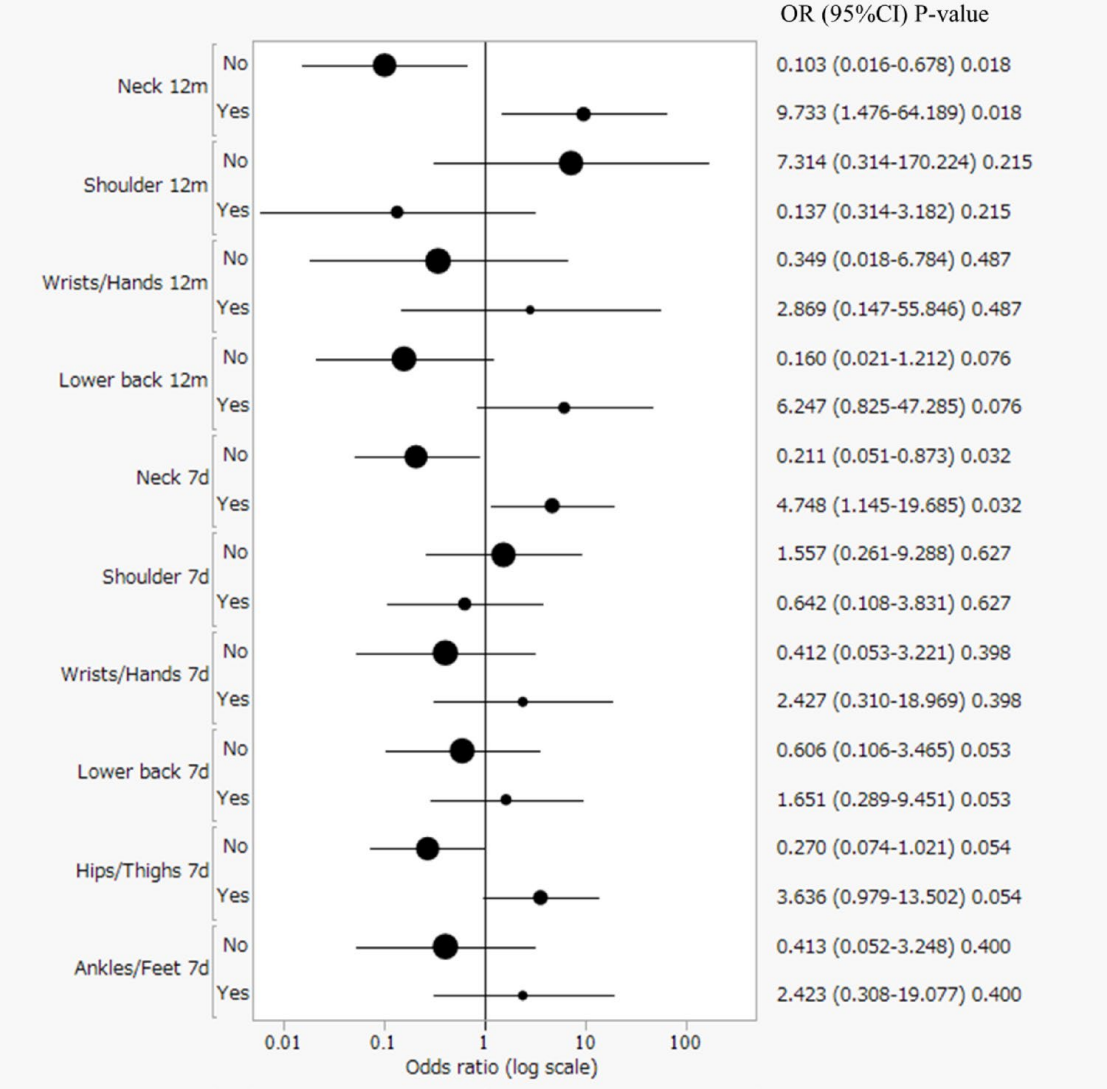
Odds ratios for non-steroidal anti-inflammatory drug (NSAID) use by anatomical site for chronic and acute musculoskeletal disorders (MSDs). Odds ratios are presented on a log scale. The analysis was conducted separately for chronic musculoskeletal disorders (MSDs) (> 12 months) and acute MSDs (< 7 days). The labels on the y-axis, “12m” and “7d”, correspond to chronic MSDs (symptoms lasting more than 12 months) and acute MSDs (symptoms experienced within the past 7 days), respectively. Each point represents the odds ratio (OR), with horizontal lines indicating the 95% confidence interval (CI). The size of the dots reflects the sample size. The numerical values on the right indicate the OR, 95% CI, and the corresponding p-value. Notably, surgeons with either chronic or acute neck pain had a significantly higher likelihood of using non-steroidal anti-inflammatory drugs (NSAIDs)

Table 2 shows the prevalence of pain after surgery. Pain severity for the neck, shoulders, and low back during open and laparoscopic surgery was significantly correlated (*P* < 0.05). An odds ratio analysis of the use of NSAIDs by anatomical site indicated that it was significantly more common among surgeons with neck pain. Specifically, the odds of taking NSAIDs were significantly higher for surgeons reporting chronic neck pain (OR 9.73, 95% CI 1.48–64.19, *P* = 0.018) as well as for those reporting acute neck pain (OR 4.75, 95% CI 1.15–19.69, *P* = 0.032) (Fig. 2).

Next, we analyzed associations between the prevalence of MSDs and various work-related and background factors. There was no significant association between total weekly operating time (> 10 h) and chronic MSDs (*P* = 0.291) or acute MSDs (*P* = 0.339). Similarly, the duration of the longest individual case (> 10 h) was not significantly associated with chronic MSDs (*P* = 0.728) or acute MSDs (*P* = 0.416). We found no significant associations between the prevalence of chronic or acute MSDs and surgeon background factors, including work experience, affiliation, BMI, height, gender, or age group (all *P* > 0.05). Similarly, reported regular exercise habits were not associated with either chronic or acute MSDs. While overall prevalence did not differ by gender, an analysis of specific sites revealed a significant association between acute knee issues and female surgeons (Pearson’s χ^2^ = 4.99, *P* = 0.026); however, this was based on a single case and requires cautious interpretation.

**Fig. 3:**
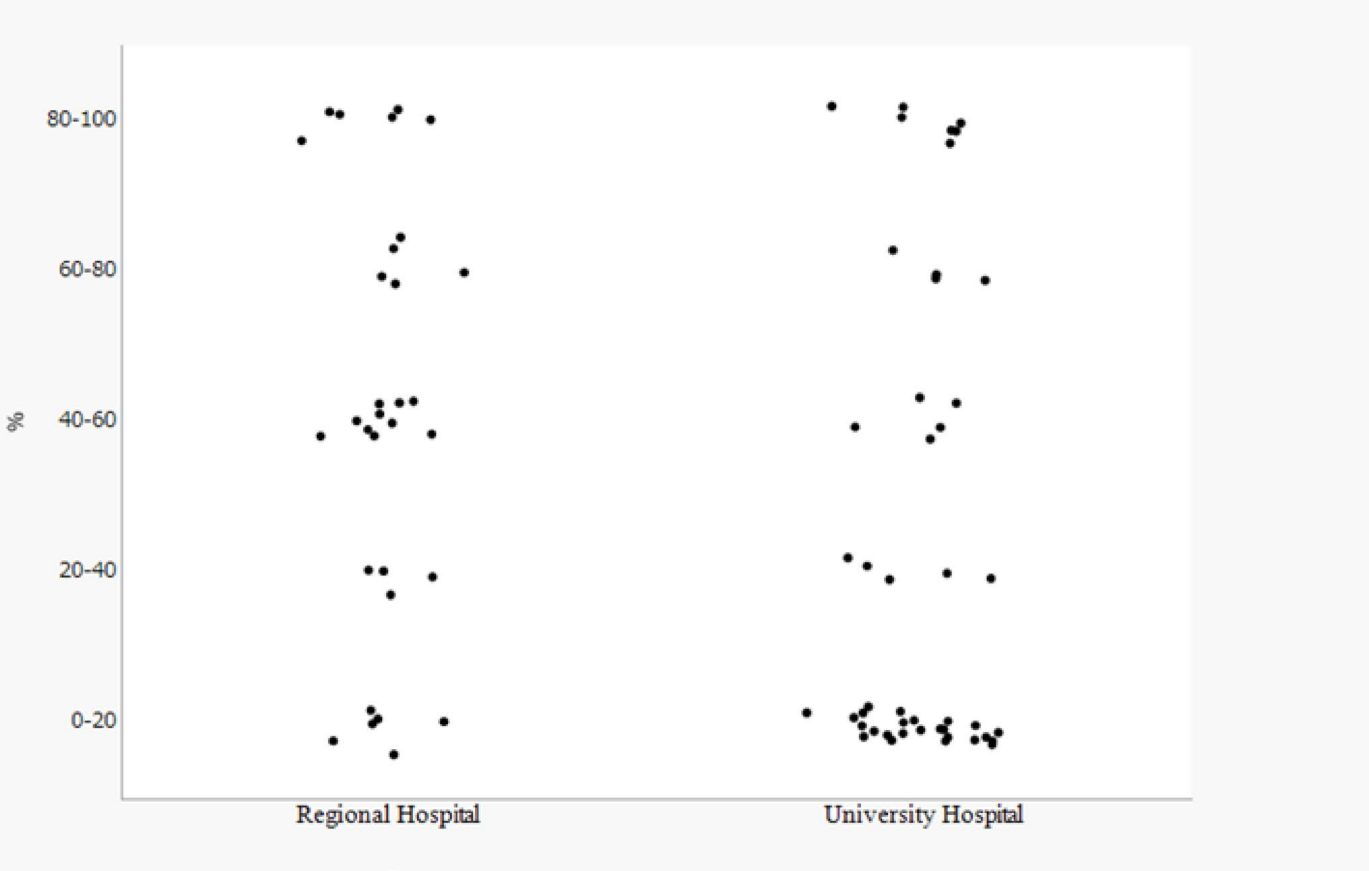
Ratio of minimally invasive surgery (MIS) to total weekly operating hours in regional hospitals vs. the university hospital. The proportion of weekly operating hours devoted to minimally invasive surgery was significantly lower for the university hospital vs. the regional hospitals (Pearson χ2 = 11.111, *P* = 0.025)

**Fig. 4:**
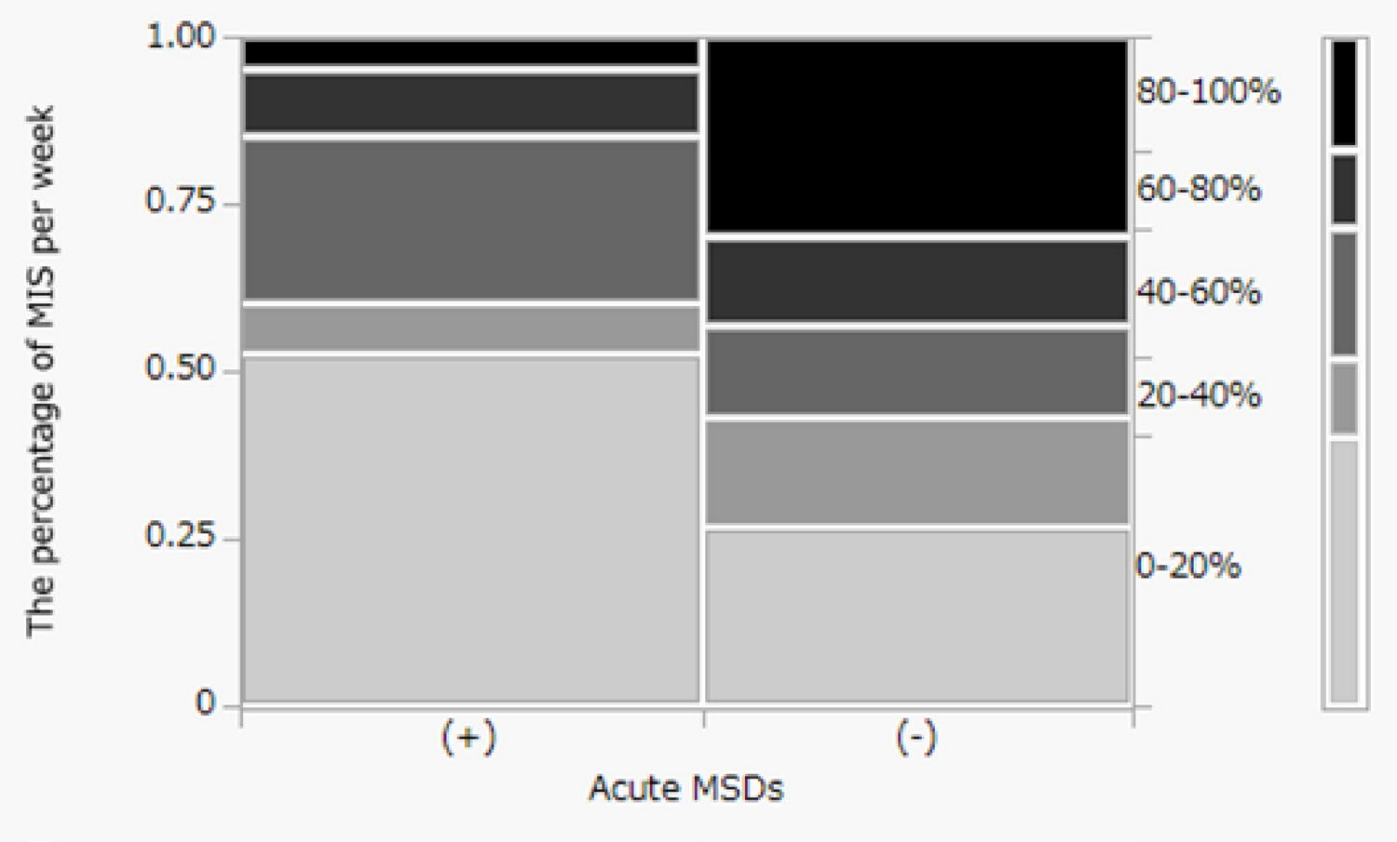
Distribution of surgeons with and those without acute musculoskeletal disorders (MSDs) across different categories of weekly minimally invasive surgery (MIS) load. The mosaic plot illustrates the distribution of surgeons with and those without acute musculoskeletal disorders (MSDs) across different categories of weekly minimally invasive surgery (MIS) load. The width of the columns is proportional to the number of surgeons with (+) or without (-) acute MSDs. The height of the segments within each column represents the proportion of surgeons in each MIS category. A significant association was identified by Fisher’s exact test between the proportion of MIS performed and the prevalence of acute MSDs (*P* = 0.011). Acute MSDs were most prevalent among surgeons performing 0–20% MIS (67.7%) and 40–60% MIS (66.7%), and least prevalent among those performing 80–100% MIS (15.4%)

We also examined the relationship between the proportion of weekly surgical time dedicated to MIS and MSDs. The proportion of MIS was significantly lower at the university hospital than at the regional hospitals (*P* = 0.025) (Fig. 3). No significant association was found by Fisher’s exact test between the proportion of MIS and chronic MSDs (*P* = 0.378). However, a significant association was found for acute MSDs (*P* = 0.011). Notably, the prevalence of acute MSDs was highest in the lowest MIS category (0–20%: 67.7%) and the moderate MIS category (40–60%: 66.7%), while it was lowest in the highest MIS category (80–100%: 15.4%) (Fig. 4).

## Discussion

The prevalence of MSDs among general surgeons in Japan remains insufficiently documented, with limited studies addressing this occupational health issue. To date, only one systematic investigation by Owada et al. has examined MSDs among Japanese surgeons [12]. Our study expands upon this by identifying a significant association between locations of pain, both chronic (≥ 12 months) and acute (≤ 7 days), and the use of NSAIDs. This is the first study to report a high prevalence of NSAID consumption among surgeons experiencing neck pain, highlighting a previously unrecognized coping mechanism.

Our study revealed the significant prevalence of MSDs among general surgeons, with specific predilections for the neck, shoulders, and lower back, and the notable association between MSDs and factors such as prolonged surgical duration, work-related stress, and psychological distress. The response rate of our survey (56.6%) was comparable to that of the previous study (60.9%), and the demographic characteristics, including mean surgical experience (17 vs. 16 years), were similar. This alignment enables meaningful comparisons. Our findings indicate that 64.9% of surgeons experienced musculoskeletal pain within the past month, closely matching the previous study’s 65.1%. The 1-month prevalence of MSDs in open and laparoscopic surgery was 45.5% and 44.2%, respectively, consistent with previous estimates (44.3% and 36.9%). However, our study found notably higher lifetime prevalence rates (open surgery: 74.0%, laparoscopic surgery: 63.3%) than previous studies (57.5% and 50.0%). This discrepancy may reflect the prevalence of minimally invasive surgery, the distribution of target diseases and the ageing of surgeons in Japan. Moreover, the psychological impact of MSDs was comparable across studies, with similar rates of mental distress (57.1% vs. 61.9%) and work absence (5.2% vs. 6.6%). Notably, chronic and acute MSDs were significantly correlated with the use of NSAIDs and mental distress. Unlike prior findings, our study did not demonstrate an age-related increase in MSD prevalence, chronic pain, or work absence, possibly because of the differences in workplace distribution among respondents. Understanding these key aspects is crucial for developing targeted interventions to mitigate the burden of MSDs in this population.

Our findings are broadly consistent with the international literature, which reports a high prevalence of work-related MSDs among surgeons globally. For instance, studies from North America and Europe have documented prevalence rates ranging from approximately 60% to over 90% across various surgical specialties [14–16]. While direct comparisons are made difficult by differences in survey methodologies and definitions, our observed prevalence of acute (51.9%) and chronic (37.7%) MSDs places Japanese surgeons within this high-risk professional landscape. The particularly high rate of acute symptoms may reflect the demanding work schedules and limited ergonomic interventions reported in the Japanese surgical environment, highlighting a shared global challenge that may require locally tailored solutions.

The significant prevalence of MSDs among general surgeons, particularly in the neck, shoulders, and lower back, aligns with previous studies that have documented high rates of musculoskeletal complaints in surgical populations [12]. These findings highlight the physically demanding nature of surgical practice, which often involves prolonged static postures, repetitive movements, and the use of specialized equipment such as surgical loupes and headlights [17, 18]. The high incidence of neck pain identified in our study may be attributed to the constrained and often awkward neck positions adopted during surgical procedures, especially open surgeries, which require direct visualization of the surgical field [19]. The odds ratio analysis reveals a nuanced relationship between the use of NSAIDs and MSDs across different anatomical sites. This study is the first to document a strong association between neck pain and NSAID use among surgeons. This finding suggests that MSDs influence pain management behaviors among surgical professionals. The neck and shoulder regions have the most significant associations with NSAID use, both for chronic (> 12 months) and acute (< 7 days) pain. The elevated odds ratios correlate with the ergonomic challenges inherent in surgical practice, particularly prolonged neck flexion and awkward cervical postures [17, 18].

Meltzer et al. utilized wearable sensors to quantify ergonomic risk, reporting that surgeons spend 65% of their operative time in high-risk cervical postures [18]. Similarly, Yang et al. demonstrated that prolonged non-neutral neck positions contribute to significant musculoskeletal strain [20]. In the Japanese context, Owada et al. found neck-related MSDs were more prevalent in association with open surgery (44.3%) than with laparoscopic procedures (28.2%) [12]. For chronic pain, the neck region shows the highest odds ratio, indicating that surgeons experiencing persistent neck discomfort are significantly more likely to rely on NSAIDs for pain management. The shoulder, which moves in conjunction with the neck, has a similar pattern, which may reflect the combined biomechanical stress from sustained posture and repetitive motion. The acute pain analysis reveals a slightly different distribution of odds ratios, with lower back and hip/thigh regions showing notable increases. This variation suggests that short-term and long-term musculoskeletal stress may manifest differently among surgical professionals. LaMonica et al. suggested that providing real-time ergonomic feedback through wearable technology could mitigate MSD risks [19]. Consequently, these findings underscore the critical need for structured ergonomic guidelines, such as the Surgical Ergonomics Recommendations by the American College of Surgeons [21], to improve working conditions and potentially extend surgical careers.

Our study further explored how the volume of different surgical approaches might influence MSDs by examining the proportion of weekly surgical time dedicated to MIS. A noteworthy finding was the significant and complex non-linear association between the proportion of MIS and the prevalence of acute MSDs (*P* = 0.012). This finding contrasts with our analysis showing no significant association between the proportion of MIS and chronic MSDs (*P* = 0.449), suggesting that the relative volume of MIS undertaken may have a different impact on acute musculoskeletal strain than on the development or persistence of long-term conditions. Specifically, the prevalence of acute MSDs was highest among surgeons performing a very low proportion of MIS (0–20% of weekly surgical time: 67.7% reported acute MSDs) and those in a moderate MIS category (40–60%: 66.7%), whereas surgeons with the highest proportion of MIS (80–100%) reported the lowest rates of acute MSDs (15.4%). The high rate of acute MSDs in the group with the lowest MIS proportion, likely performing predominantly open surgery, is consistent with the recognized physical demands of open procedures, such as the prolonged static postures and awkward neck positions often required for direct visualization [18, 20]. The elevated prevalence of acute MSDs in the moderate MIS group (40–60%) is more intricate. It could be hypothesized that surgeons in this category frequently alternate between open and MIS techniques, thereby facing the ergonomic challenges of both. Alternatively, they might be undertaking complex MIS procedures without having achieved the volume-related efficiencies or ergonomic adaptations of surgeons performing a high proportion of MIS procedures. The lower incidence of acute MSDs observed in surgeons performing the highest proportion of MIS (80–100%) warrants careful interpretation. This finding may be partly attributable to a “survivor effect,” wherein surgeons less tolerant to the specific strains of MIS do not maintain such high volumes. Furthermore, surgeons who perform MIS almost exclusively may benefit from a more consistent and ergonomically optimized operating room setup and workflow than those who frequently switch between open and laparoscopic modalities. This is an important consideration, even as it is acknowledged that MIS itself presents distinct ergonomic risks, such as those related to monitor use and instrument manipulation [3]. These varying rates of acute MSDs across MIS categories become especially pertinent when considering our finding that the university hospital had a significantly lower proportion of MIS procedures performed weekly than the regional hospitals. This difference in practice patterns may mean that surgeons at the university hospital, potentially more represented in the lower MIS proportion categories, could experience a different profile of acute MSDs risks. Conversely, while regional hospitals may have more surgeons in the highest MIS category (who reported fewer acute MSDs), they would also have surgeons in the moderate MIS categories, which also showed a high prevalence of acute MSDs. It is important to note that the Chi-Square analysis for the association between MIS proportion and acute MSDs indicated that 20% of cells had an expected frequency below 5, warranting some caution in the interpretation of the P-value’s precision. Further research could be beneficial to explore not only the proportion of MIS procedures, but also specific procedure types, operative duration within MIS, and the impact of ergonomic interventions in mitigating these observed risks for acute MSDs.

The broader implications of our findings extend beyond the individual surgeons affected by MSDs. The high prevalence of MSDs among general surgeons in Japan may have significant consequences for the healthcare system, potentially leading to a shortage of experienced surgeons and compromising the quality of surgical care [1]. Given the aging population [22] and the increasing demand for surgical interventions [23, 24], it is crucial to ensure the long-term sustainability of the surgical workforce. Implementing ergonomic interventions, promoting work-life balance, and providing psychological support are essential steps to safeguard the health and well-being of surgeons, ultimately benefiting patients and healthcare institutions. Our findings underscore the need for national guidelines and preventive strategies tailored to the specific needs of Japanese surgeons, considering the unique cultural and organizational factors that may contribute to MSDs in this population.

Several methodological limitations must be acknowledged. First, the cross-sectional design of this study allows for the identification of associations but precludes the establishment of causal relationships. Second, data collection was confined to a single university hospital network, potentially limiting the generalizability of our findings. Third, the survey was conducted during a specific period (July to September, 2024). While major seasonal fluctuations in surgical caseloads are not typical in our network, we cannot rule out potential seasonal effects on musculoskeletal symptoms, which may affect the generalizability of the findings. Fourth, while our sample included a relatively high proportion of female surgeons (16.9%) compared with the national average (8.3% as reported by the Ministry of Health, Labor, and Welfare), it remains insufficient to assess gender-specific MSDs risks comprehensively. Fifth, institutional composition (60% university hospitals, 40% regional hospitals) and variations in minimally invasive surgery adoption may have influenced our results. Differences in surgical techniques, procedural duration, and specialty distribution across institutions represent additional confounding variables. Furthermore, this study could not analyze the ergonomic impact of robotic-assisted surgery separately. While robotic platforms are being introduced in Japan, their adoption within our network during the survey period was limited, preventing a meaningful sub-analysis. Future research should focus on the specific ergonomic risks and benefits of robotic surgery as its use grows. Sixth, our sample predominantly comprised mid-career and senior surgeons (median experience: 17 years), limiting our ability to characterize the prevalence of MSDs among early-career surgeons, who may face distinct ergonomic challenges with evolving training environments and case volumes. Seventh, the inherent limitations of survey-based research, including response bias (social desirability effects), selection bias, and recall bias, must be considered. Finally, our reliance on binary response options may have oversimplified the complexity of MSDs, failing to capture the progressive nature of musculoskeletal strain over time. Future research should incorporate mixed methods approaches, including graduated response scales, qualitative assessments, and objective ergonomic measurements, to provide a more comprehensive understanding of work-related MSDs among surgeons.

The high prevalence of MSDs identified in our study underscores the urgent need for preventive strategies. While our research was designed to define the scope of the problem, these findings highlight the critical importance of exploring solutions. Therefore, we strongly recommend that future research focuses on evaluating specific, practical interventions tailored to the surgical environment. This could include studies on the ergonomic benefits of intraoperative micro-breaks [25], the feasibility of seated operating [26], the impact of optimized operating room design [27], and the potential of wearable ergonomic aids [28], such as assist suits, to mitigate physical fatigue on the neck, shoulders, and back. Translating the foundational data from descriptive surveys like ours into evidence-based interventions is the essential next step to protect the well-being and ensure the sustainability of the surgical workforce.

## Conclusion

This study highlights the significant burden of MSDs on general surgeons in Japan and emphasizes the necessity for targeted interventions. A substantial proportion of surgeons reported chronic symptoms and functional impairment, with MSDs adversely affecting their surgical practice, daily activities, and psychological well-being. Many of these surgeons relied on NSAIDs for symptom management, with neck pain being particularly associated with increased NSAID use. These findings suggest that ergonomic improvements and awareness programs are essential to reduce the risk of MSDs in surgical practice.

**Table 1:** Responses from surgeons about their basic characteristics and work-related factors

Characteristics		
	**Median**	**IQR**
**Height (cm)**	170	165-175
**Weight (kg)**	67	59.5-76.0
**BMI**	23	22-25
**Gender**	***N*** **=77**	**%**
Male	63	81.8
Female	13	16.9
NA	1	1.3
**Age, (years)**	***N*** **=77**	**%**
< 29	1	1.3
30-39	22	28.6
40-49	32	41.6
50-59	19	24.7
>60	3	3.9
**Work experience (years)**	***N*** **=77**	**%**
<10	10	13.0
10-20	30	39.0
20-30	20	26.0
>30	12	15.5
Missing	5	6.5
**Affiliation**	***N*** **=77**	**%**
Regional hospital	31	40.3
University hospital	46	59.7
Upper GI team	6	7.8
Colorectal team	9	11.7
HPB team	13	16.9
Breast team	3	3.9
Transplant team	4	5.2
Resident	11	14.3
**Weekly OT (hours)**	***N*** **=77**	%
<1	8	10.4
1-2	3	3.9
2-3	4	5.2
3-4	6	7.8
4-5	0	0
5-6	13	16.9
6-7	0	0
7-8	8	10.4
8-9	4	5.2
9-10	4	5.2
>10	27	35.1
**Longest OT of the week (hours)**	***N*** **=77**	**%**
<1	4	5.2
1-2	0	0
2-3	9	11.7
3-4	13	16.9
4-5	0	0
5-6	14	18.2
6-7	0	0
7-8	9	11.7
8-9	7	9.1
9-10	1	1.3
>10	19	24.7
NA	1	1.3
**Weekly ratio of MIS**	***N*** **=77**	**%**
<20%	31	40.3
20-40%	9	11.7
40-60%	15	19.5
60-80%	9	11.7
>80%	13	16.9

NA: not applicable, IQR: interquartile range, HPB: hepato-pancreato-biliary, OT: Operation time, MIS: Minimal invasive surgery

**Table 2:** Prevalence of musculoskeletal pain, psychological distress, and leave of absence

	*N*=77	%
**Pain after surgery**		
Last month prevalence		
Open surgery	35	45.5
Laparoscopic surgery	34	44.2
Lifetime prevalence		
Open surgery	57	74.0
Laparoscopic surgery	49	63.6
**Chronic pain**	26	33.8
**Psychological distress related to pain**	44	57.1
**Leave of absence**	4	5.2

## Supplementary Information

Below is the link to the electronic supplementary material.

Supplementary file1

## Data Availability

The data that support the findings of this study are not publicly available because they contain information that could potentially identify research participants. The data are available from the corresponding author, H.S., upon reasonable request.
