# Supplementary material for: Current status and characteristics of work-related musculoskeletal disorders among general surgeons in japan: a cross-sectional survey at a university hospital and its affiliated regional hospitals
Source: Surg Today. 2025 Nov 14;56(5):697–706. doi: 10.1007/s00595-025-03174-z (PMC13090198; doi:10.1007/s00595-025-03174-z)
Supplement: Supplementary file 1 — Supplementary file1 [file 595_2025_3174_MOESM1_ESM.docx]

**Supplementary Materials**

**Supplementary Table 1.** Questionnaire on background, work-related factors, and musculoskeletal disorders

| **Background** |  |
| --- | --- |
| What year did you or will you complete medical school? | NR |
| What is your age group? | SA |
| What is your gender?! | SA |
| What is your height in centimeters? | NR |
| What is your weight in kilograms? | NR |
| What is your body mass index (BMI)? | NR |
| **Work-related** |  |
| Where do you work? If you work at a university hospital, please select your group. | SA |
| How many hours per week in total do you perform surgery? | SA |
| What is the longest operation that you would perform in a week? | SA |
| What percentage of minimally invasive surgical procedures would you perform per week? | SA |
| **Musculoskeletal disorders (MSDs)** |  |
| Location of the MSDs (Supplementary Figure 1) | SA |
| Have MSDs had a negative impact on surgical practice? | NR |
| Have MSDs had a negative impact on your daily life? | SA |
| Have you ever taken a leave of absence from surgery due to MSDs caused by performing surgery? | SA |
| Have you seen a physician for MSDs? | SA |
| How often do you take oral NSAIDs for MSDs? | SA |
| □ least once a week, □ 1-3 times a month, □ once every 2-6 months, □ once a year, □ never |  |
| Do you consider MSDs to be relevant to your work? | NR |
| Have you ever had chronic pain in your neck, shoulders, or lower back caused by performing surgery? *Chronic pain is defined as repeated pain or continuous pain for more than a month | SA |
| How much psychological distress or anxiety was caused by pain in your neck, shoulders or lower back from the surgery you ghave performed? 1 (none), 2(mild), 3(moderate), 4 (severe) | SA |
| Have you ever had pain in your neck, shoulders, or lower back after performing open surgery?  1 (none), 2(mild), 3(moderate), 4 (severe) | SA |
| Do you currently have pain in your neck, shoulders or lower back after performing open surgery (within the past month)? 1 (none), 2(mild), 3(moderate), 4 (severe) | SA |
| Have you ever had pain in your neck, shoulders or lower back after performing laparoscopic surgery?  1 (none), 2(mild), 3(moderate), 4 (severe) | SA |
| Do you currently have pain in your neck, shoulders or lower back after performing laparoscopic surgery (within the past month)? 1 (none), 2(mild), 3(moderate), 4 (severe) | SA |

NR, numeric response; SA, single answer

**Supplementary Fig. 1**

**
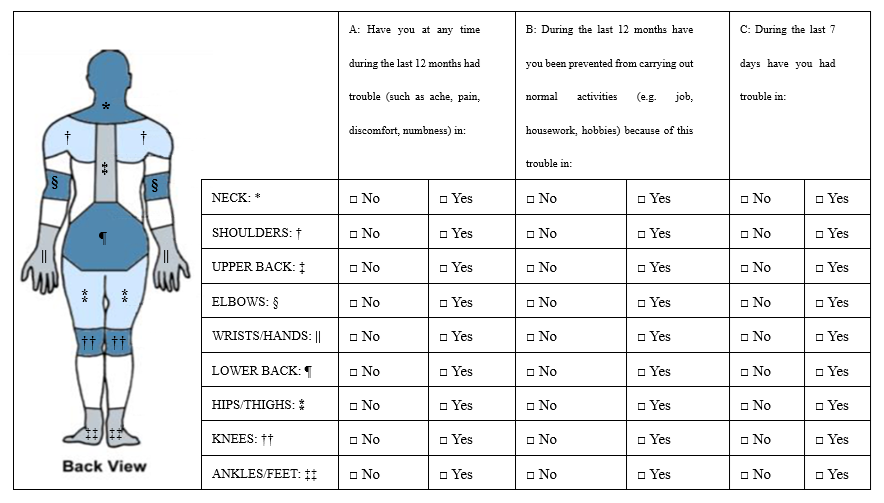
**
